# Characterization of polybacterial clinical samples using a set of group-specific broad-range primers targeting the 16S rRNA gene followed by DNA sequencing and RipSeq analysis

**DOI:** 10.1099/jmm.0.028373-0

**Published:** 2011-07

**Authors:** Øyvind Kommedal, Katrine Lekang, Nina Langeland, Harald G. Wiker

**Affiliations:** 1 Section for Microbiology and Immunology, The Gade Institute, University of Bergen, 5020 Bergen, Norway; 2 Institute of Biology, University of Bergen, 5020 Bergen, Norway; 3 Institute of Medicine, University of Bergen, 5020 Bergen, Norway; 4 Department of Microbiology, Haukeland University Hospital, 5021 Bergen, Norway

## Abstract

The standard use of a single universal broad-range PCR in direct 16S rDNA sequencing from polybacterial samples leaves the minor constituents at risk of remaining undetected because all bacterial DNA will be competing for the same reagents. In this article we introduce a set of three broad-range group-specific 16S rDNA PCRs that together cover the clinically relevant bacteria and apply them in the investigation of 25 polybacterial clinical samples. Mixed DNA chromatograms from samples containing more than one species per primer group were analysed using RipSeq Mixed (iSentio, Norway), a web-based application for the interpretation of chromatograms containing up to three different species. The group-specific PCRs reduced complexity in the resulting DNA chromatograms and made the assay more sensitive in situations with unequal species concentrations. Together this allowed for identification of a significantly higher number of bacterial species than did standard direct sequencing with a single universal primer pair and RipSeq analysis (95 vs 51). The method could improve microbiological diagnostics for important groups of patients and can be established in any laboratory with experience in direct 16S rDNA sequencing.

## Introduction

Detection and identification of bacteria directly from clinical samples by broad-range PCR targeting the 16S rRNA gene followed by DNA sequencing (direct 16S rDNA sequencing) is a valuable tool in clinical microbiology (Goldenberger *et al.*, 1997; Harris & Hartley, 2003; Petti *et al.*, 2008b). RipSeq (iSentio, Bergen, Norway) is a web-based application for the analysis of mixed DNA chromatograms that enables the use of this method also on typical polybacterial specimens such as abscesses and empyema (Hartmeyer & Justesen, 2010; Kommedal *et al.*, 2009). However, the use of a single universal first PCR limits the potential of this approach because the various bacterial DNAs in a sample will compete for the same reagents, leaving those present at the lowest concentrations at risk of remaining undetected. We have found that this will occur when the difference in concentrations exceeds 1 : 10, with some variation dependent on primer affinity for the different targets and the number of 16S rRNA gene copies in the respective bacteria. Also, the RipSeq algorithm has been validated for chromatograms containing up to three different species only (Kommedal *et al.*, 2008). The aim of this study was to increase the number of detectable species in polybacterial samples by replacing the single universal PCR with a set of group-specific broad-range PCRs.

Based on careful examination of the 16S rRNA gene we identified a semi-conserved area splitting a wide selection of human bacterial pathogens into three variant groups: group A, Gram-positive cocci in chains and non-anaerobic Gram-positive rods; group B, non-anaerobic Gram-negative bacteria; group C, *Staphylococcus* spp. and anaerobic bacteria not covered in group A. Further research allowed for the design of three group-specific broad-range primer pairs (A, B and C) each matching one of the three groups. All primer pairs amplified approximately the first 500 bp of the gene. In addition to reducing competition for reagents they also increased the theoretical number of species possible to detect in a sample using RipSeq from three to three times three. The group-specific primers were used to reinvestigate 50 clinical samples, of which 25 were known to be polymicrobial and 25 assumed to be sterile based on previous investigations and the clinical information available.

## Methods

### 

#### Primer cross-reactivity.

For each primer pair (A, B and C; see Table 1 for primer sequences) cross-reactivity with the other bacterial groups was investigated by amplifying series of mixes with a falling ratio of target to non-target DNA (1 : 1 to 1 : 1000). In PCRs with primer pair A and primer pair C cloned 16S rDNA fragments were used. In reactions with primer pair B genomic DNA was used instead due to residual *Escherichia coli* DNA from the cloning process. Cross-reactivity with human DNA was tested for each primer pair by amplifying 1000 pg, 100 pg, 10 pg, 1 pg and 0 pg of target bacterial DNA together with a constant amount of 100 ng human DNA (Promega).

**Table 1.** Complete list of primers used in this study Capital letters indicate a locked nucleic acid (LNA). Ambiguous bases: K = G or T, M = A or C, R = A or G.

Primer	Sequence (5′–3′)
**Cloning experiment**	
Forward (4F)*	ttg-gag-agt-ttg-atc-mtg-gct-c
Reverse (pH/1542r)†	aag-gag-gtg-atc-cag-ccg-ca
**Universal PCR**	
Forward (4F)	ttg-gag-agt-ttg-atc-mtg-gct-c
Reverse (pD)†	gta-tta-ccg-cgg-ctg-ctg
**Universal cycle sequencing reactions**	
Forward (Bact-8S-20)‡	aga-gtt-tga-tcm-tgg-ctc-ag
Reverse (pD)	gta-tta-ccg-cgg-ctg-ctg
**Group-specific PCRs**	
Group A-forward	-gt-tTg-atc-mtg-gct-cag-rAc
Group B-forward	-gt-tTg-atc-mtg-gct-cag-aKtg
Group C-forward	agt-ttg-atc-mtg-gct-cag-gAt
Groups A, B, C-reverse (pD)	gta-tta-ccg-cgg-ctg-ctg
**Group specific cycle sequencing reactions**	
Common reverse (534R)*	tac-cgc-ggc-tgc-tgg-cac

* Petti *et al.* (2008a).

† Edwards *et al.* (1989).

‡ Bosshard *et al.* (2002).

#### Cloning and plasmid isolation.

The 16S rRNA genes from relevant bacterial strains were amplified using primers 4F+pH/1542r (Table 1). The PCR products were cloned into pCR 2.2 TOPO vector and transformed into competent *E. coli* cells using the TOPO TA cloning kit (Invitrogen), following the manufacturer’s protocol. *E. coli* containing verified clones were cultured aerobically overnight. Plasmids were isolated from 2 ml *E. coli* culture using PureLink Quick Plasmid Miniprep kit (Invitrogen). The isolated plasmids were diluted to 10^10^ µl^−1^ and frozen at −20 °C until they were used.

#### Clinical samples.

The following polymicrobial specimens were included together with their corresponding negative controls: brain abscess (*n* = 5), bile (*n* = 2), empyema (*n* = 6), liver abscess (*n* = 3), ovarian abscess (*n* = 1), pancreas abscess (*n* = 2), para-aortic abscess (*n* = 1), retro-peritoneal abscess (*n* = 2), soft tissue abscess (*n* = 2) and spleen abscess (*n* = 1). In addition 25 presumptive negative specimens and their controls were included: various abscess or abscess-like specimens (*n* = 5), bone biopsy (*n* = 1), pericardial fluid (*n* = 3), periprosthetic tissue (*n* = 3), peritoneal fluid (*n* = 2), pleural fluid (*n* = 6), subdural haematoma (*n* = 1) and synovial fluid (*n* = 4). All samples were investigated using the three separate group-specific real-time PCRs with SYBR-green detection followed by DNA sequencing. In parallel all samples were analysed using a single universal primer pair (4F+pD, Table 1) targeting the same part of the 16S rRNA gene as the group-specific primers (the first 500 bp).

#### Pre-PCR treatment of clinical samples.

The samples had been extracted using the following protocol. Between 200 and 800 µl sample material was added to a tube containing a mixture of glass and ceramic beads (SeptiFast Lysis kit, Roche), together with 400 µl Bacterial Lysis Buffer (BLB, Roche). Eight hundred microlitres was the maximum capacity of the bead-tube, and this volume was used for liquid samples with low viscosity. For other samples 400 µl was used if available. Two hundred microlitres was the lowest volume that would still provide 400 µl supernatant for the subsequent DNA purification and was the lowest volume accepted for all specimens. A negative control containing lysis buffer and 400 µl PCR-grade water (Qiagen) was included in every batch of samples. The samples were run for 2×45 s in a FastPrep machine (Cepheid) at speed 6.5. After a short spin (15 800 ***g***, 3 min), 400 µl supernatant was transferred to a MagNa Pure Compact automated extractor (Roche) and DNA was extracted and purified using the ‘Total_NA_plasma_100_400’ programme according to the manufacturer’s instructions. The resulting 50 µl of eluate was stored at −80 °C until used.

#### PCR and PCR conditions.

PCR primers are listed in Table 1. All PCRs were performed in 25 µl reaction tubes on a real-time SmartCycler apparatus (Cepheid) with a final reaction volume of 25 µl. The universal PCR mixture consisted of 12.5 µl ExTaq SYBR master mix (TaKaRa), 0.4 µM F-primer and 0.4 µM R-primer, 8.5 µl PCR-grade water and 2 µl template. All group-specific PCR mixtures consisted of 12.5 µl ExTaq SYBR master mix, 0.8 µM F-primer and 0.4 µM R-primer, 7.5 µl PCR-grade water and 2.0 µl template. The PCR thermal profile was identical for all reactions and included an initial polymerase activation step of 10 s at 95 °C followed by 40 cycles of 15 s at 95 °C, 10 s at 64 °C and 20 s at 72 °C.

#### Negative controls.

For all PCRs every sample was run in parallel with its negative extraction and purification control. For the universal PCR a sample was defined as positive if the fluorescence threshold value (*C*_t_) was reached three or more cycles ahead of the negative control. For the group-specific PCRs the negative control that became positive first for a given sample defined the cut-off for positivity for all three group-specific PCRs for that sample (i.e. three or more cycles before the earliest negative control).

#### Sequencing.

The amplicons from the positive PCRs were collected by centrifugation from the SmartCycler reaction tubes into a 1.5 ml Eppendorf tube and cleaned up using the ExoSAP-IT enzymic degradation kit (Affymetrix). Primers used in the cycle sequencing reactions are listed in Table 1. Sequencing was performed in a core facility using the ABI PRISM 1.1 Big-Dye sequencing kit and a 3730 DNA Analyser (Applied Biosystems).

#### Interpretation of chromatograms.

Mixed DNA chromatograms were analysed using the RipSeq Mixed web application (http://www.ripseq.com/login/login.aspx). The RipSeq Mixed algorithm searches against the ‘16S human pathogen iSentio’ database currently containing about 1500 reference sequences. The definition of a positive identification with the RipSeq program has been described previously (Kommedal *et al.*, 2008). Non-mixed chromatograms were analysed with both RipSeq and a standard blast search against the GenBank database (http://blast.ncbi.nlm.nih.gov). For the blast searches interpretation criteria given by the Clinical and Laboratory Standards Institute were followed (Petti *et al.*, 2008a).

## Results

### Validation of new primers for PCR

The group-specific primers were aligned against 160 high-quality GenBank reference sequences representing all 129 different genera present in the ‘16S human pathogen iSentio’ database.

The distribution of genera among the different primer pairs was as follows: primer pair A, 42 genera [Gram-positive cocci in chains (including some anaerobic species) and non-anaerobic Gram-positive rods (including the aerotolerant genera *Actinomyces* and *Propionibacterium*)]; primer pair B, 46 genera (non-anaerobic Gram-negative bacteria); primer pair C, 41 genera (*Staphylococcus* spp. and anaerobic bacteria except those covered for by primer A). This distribution is to be viewed as a rule of thumb, and exceptions exist in particular for groups A and C. A more detailed overview is provided in Supplementary Table S1, available with the online version of this paper.

The highest tendency for cross-reactivity was between primer pair A (PCR ‘A’) and bacterial group C and between primer pair C (PCR ‘C’) and bacterial group A. For these combinations a 1000-fold higher copy number of non-target DNA was needed to reach equally high peaks as the target DNA in the resulting chromatograms. For primer pair B (PCR ‘B’) co-amplification of group ‘A’ and ‘C’ DNA was not detectable even when present at a 1000-fold higher concentration than group ‘B’ target DNA (Supplementary Table S2). No cross-reactivity with human DNA was observed with any of the primer pairs. In the solutions with 100 ng human DNA and no added bacterial DNA, only background bacterial DNA from the reagents was amplified.

### Clinical samples

The polybacterial specimens contained high levels of bacterial DNA, and with the universal PCR the *C*_t_ distance between a sample and the corresponding negative control ranged from −20 to −8 cycles. For the specimens positive by PCR ‘A’ the *C*_t_ distance to the negative control ranged from −17 to −4 cycles. For the specimens negative by PCR ‘A’ the *C*_t_ distance ranged from −2.4 to +3.9. The corresponding intervals for PCR ‘B’ were −16 to −7 and −2.8 to +0.9 and for PCR ‘C’ −17 to −6 and −2.0 to +4.4. (− indicates before the negative control and+indicates after.)

Eighty per cent of the samples were affected by antibiotics administrated prior to specimen collection, and both standard direct 16S rDNA sequencing and direct sequencing using group-specific primers detected a higher number of bacteria than did culture. In total 37 species were found by culture, 51 by standard direct sequencing and 95 by the group-specific primers. A detailed comparison between these results is given in Table 2. All bacteria identified with the standard universal primer pair were also detected with the group-specific primers. For six samples the two different sequencing protocols yielded identical results. In the remaining 19 samples a total of 44 additional bacteria were found by the group-specific primers, ranging from one to five species per sample. Only 32 out of the 95 bacteria identified by group-specific direct sequencing were cultivable. Four samples contained one or more isolates found exclusively by culture. These were a strain of *Staphylococcus haemolyticus* isolated from sample 7, scarce growth of a coagulase-negative Staphylococcus together with a diphtheroid in sample 23 and two colonies of *Streptococcus parasanguinis* in sample 24. In addition an isolate of *Klebsiella* sp. from sample 6 was not possible to read from chromatogram 6B. Some of these isolates might represent sample contamination, but others are probably true findings that have not been detected by sequencing either because they had to compete for reagents with a more dominant species belonging to the same primer group or because direct sequencing can have lower sensitivity than culture in samples with living bacteria.

**Table 2.** Comparison of results obtained by culture, direct sequencing with a single universal primer pair and direct sequencing with the group-specific primer pairs

ID	Sample type	Culture*	Universal primer	Group-specific primers	Antibiotic†
1	Abscess brain			*Fusobacterium nucleatum*	CX, RI
				*Gemella haemolysans/morbillorum*	
			*Parvimonas* sp.‡	*Parvimonas *sp.	
		*Streptococcus constellatus*	*Streptococcus intermedius*	*S. intermedius*	
2	Abscess brain			*F. nucleatum*	CX, RI
		*S. constellatus*	*S. intermedius*	*S. intermedius*	
3	Abscess brain			*Aggregatibacter aphrophilus*	CX, MZ
				*Eikenella *sp.	
			*F. nucleatum*‡	*F. nucleatum*	
				*F. nucleatum *subsp.* polymorphum*	
				*Neisseria elongata*	
		*Peptostreptococcus* sp.	*Parvimonas micra*	*P. micra*	
		*Streptococcus milleri* group	*S. intermedius*	*S. intermedius*	
4	Abscess brain	No growth		*Actinomyces meyeri*	CT, MZ
				*Campylobacter gracilis*	
			*F. nucleatum*‡	*F. nucleatum*	
				*Prevotella pleuritidis*	
			*S. intermedius*	*S. intermedius*	
5	Abscess brain	*A. meyeri*		*A. meyeri*	–
			*C. gracilis*	*C. gracilis*	
				*Eikenella *sp.	
		*F. nucleatum*	*Fusobacterium *sp.	*Fusobacterium *sp.	
			*Peptostreptococcus *sp.	*Peptostreptococcus* sp.	
6	Abscess liver	*E. coli*	*E. coli/Shigella* spp.	*E. coli/Shigella* spp.	AM
		*Enterococcus faecium*	*E. faecium/durans*	*E. faecium/durans*	
		*Klebsiella* sp.			
				*Lactobacillus rhamnosus*	
		*Staphylococcus haemolyticus*		*S. haemolyticus*	
7	Abscess liver			*G. haemolysans*	PT
			*Prevotella melaninogenica*	*P. melaninogenica*	
			*Streptococcus mitis* group	*S. mitis *group	
				*Streptococcus parasanguinis*	
		*S. haemolyticus*			
8	Abscess liver§	No growth	*Clostridium perfringens*	*C. perfringens*	CT
			*E. coli/Shigella* spp.	*E. coli/Shigella* spp.	
9	Abscess muscle			*Eikenella corrodens*	–
			*F. nucleatum*‡	*F. nucleatum*	
				*P. micra*	
				*Porphyromonas circumdentaria*	
			*Porphyromonas endodontalis*	*P. endodontalis*	
		*Streptococcusmilleri* group		*S. constellatus/intermedius*	
10	Abscess ovarian§	*F. nucleatum*	*F. nucleatum*	*F. nucleatum*	–
		*Peptostreptococcus* sp.	*Parvimonas *sp.	*Parvimonas *sp.	
11	Abscess pancreas	*Bacteroides fragilis*	*B. fragilis*	*B. fragilis*	IP
		*E. faecium*		*E. faecium*	
12	Abscess pancreas			*A. meyeri*	IP
				*Campylobacter concisus*	
			*P. melaninogenica*	*P. melaninogenica/histicola*	
			*Prevotella histicola*		
		CNS	*Staphylococcus epidermidis*	*S. epidermidis*	
13	Abscess para-aortic			*Corynebacterium appendicis*	–
		Diphtheroid	*Corynebacterium tuberculostearicum*	*C. tuberculostearicum*	
		CNS	*S. haemolyticus*	*S. haemolyticus*	
14	Abscess psoas	No growth	*Enterobacter hormaechei*	*E. hormaechei*	MP
				*E. faecium*	
15	Abscess spleen	*Enterococcus *sp.	*Enterococcus casseliflavus/gallinarum*‡	*E. casseliflavus/gallinarum*	Unknown
				*E. coli/Shigella *spp.	
			*E. faecalis*	*E. faecalis*	
				*F. nucleatum*	
				*S. anginosus*	
		*Staphylococcus epidermidis*		*S. epidermidis*	
16	Abscess subcut.§	*E. faecium*	*E. faecium*	*E. faecium*	CI, OX
		*Finegoldia magna*	*F. magna*	*F. magna*	
17	Abscess retro-peritoneal§	*E. faecium*	*E. faecium/hirae*	*E. faecium/hirae*	CI, MZ, LZ
			*Klebsiella pneumoniae*	*K. pneumoniae*	
18	Bile§	*E. coli*	*E. coli/Shigella* spp.	*E. coli/Shigella* spp.	CT, MZ
			*F. nucleatum*	*F. nucleatum*	
19	Bile			*E. caccae*	IP
		*Enterococcus* sp.	*E. casseliflavus/gallinarum*	*E. casseliflavus/gallinarum*	
				*E. faecalis*	
		CNS	*S. epidermidis*	*S. epidermidis*	
20	Empyema	No growth	*P. micra*‡	*P. micra*	PT
				*Prevotella nigrescens*	
			*Peptostreptococcus stomatis*	*P. stomatis*	
			*S. anginosus*	*S. anginosus*	
21	Empyema§		*C. gracilis*	*C. gracilis*	CT, GE, PC
			*Fusobacterium *sp.	*Fusobacterium *sp.	
		*S. intermedius*	*S. intermedius*	*S. intermedius*	
22	Empyema		*F. nucleatum*	*F. nucleatum*	CT, PC
				*C. gracilis*	
			*P. micra*	*P. micra*	
		*S. intermedius*	*S. intermedius*	*S. intermedius*	
23	Empyema			*C. gracilis*	CI, CL
				*F. nucleatum*	
				*Haemophilus parainfluenzae*	
			*P. melaninogenica*‡	*P. melaninogenica*	
				*P. micra*	
		*S. constellatus*		*S. constellatus*	
		diphtheroid			
		CNS			
24	Empyema	*C. gracilis*		*C. gracilis*	PC
		*E. corrodens*		*E. corrodens*	
				*P. micra*	
			*Prevotella pleuritidis*	*P. pleuritidis*	
				*S. intermedius*	
		*S. parasanguinis*			
25	Empyema	*Prevotella *sp.	*P. nigrescens*	*P. nigrescens*	CT
			*Prevotella veroralis*	*P. veroralis *	
		Anaerobic Gram+ve rod		*Dialister invisus*	
				*Dialister micraerophilus*	
				*P. micra*	

* CNS, coagulase negative Staphylococcus.

† Antibiotics: –, no treatment; AM, amoxicillin; CI, ciprofloxacin; CL, clindamycin; CT, cefotaxime; CX, ceftriaxone; GE, gentamicin; IP, imipenem; LZ, linezolid; MP, meropenem; MZ, metronidazole; OX, oxacillin; PC, penicillin-G; PT, piperacillin-tazobactam; RI, rifampicin; VA, vancomycin.

‡ Chromatogram too complex to allow for complete analysis. Only dominant peaks included.

§ Samples with identical results for standard direct sequencing and group-specific direct sequencing.

The detailed results from the group-specific PCRs for the polybacterial specimens are given in Table 3. Four cases of cross-reactivity between primer groups were observed. In sample 7, a group A species was detected as low secondary peaks in chromatogram 7C. In both samples 11 and 12 a species belonging to group C was also detectable as the lower peaks in the A chromatograms. In sample 16, *Enterococcus faecium* and *Finegoldia magna* were amplified by both PCR ‘A’ and ‘C’, but PCR ‘A’ became positive 8.6 cycles prior to PCR ‘C’. Three of the group-specific chromatograms were found to be too complex to be completely resolved, indicating that these samples still might contain one or more unidentifiable species.

**Table 3.** Detailed results for group-specific direct 16S sequencing from clinical samples Species in parenthesis (*n* = 5) indicates cross-reactivity between primer groups.

ID	Sample type	Primer pair A	Primer pair B	Primer pair C
1	Abscess brain	*Gemella haemolysans/morbillorum*	*–*	*Fusobacterium nucleatum*
		*Parvimonas *sp.		
		Streptococcus intermedius*		
2	Abscess brain	*S. intermedius*	*–*	*F. nucleatum*
3	Abscess brain	*Parvimonas micra*	*Aggregatibacter aphrophilus*†	*F. nucleatum*
		*S. intermedius*	*Eikenella corrodens*	*F. nucleatum *subsp.* polymorphum*
			*Neisseria elongata*	
4	Abscess brain	*Actinomyces meyeri*	*Campylobacter gracilis*	*F. nucleatum*
		*S. intermedius*		*Prevotella pleuritidis*
5	Abscess brain	*A. meyeri*	*C. gracilis*	*Fusobacterium *sp.
		*Peptostreptococcus *sp.	*Eikenella *sp.	
6	Abscess liver	*Enterococcus faecium/durans*	*Escherichia coli/Shigella *spp*.*†	*Lactobacillus rhamnosus*
				*Staphylococcus haemolyticus*
7	Abscess liver	*G. haemolysans*	*–*	*Prevotella melaninogenica*
		*Streptococcus mitis *group		(*S. parasanguinis*)
		*Streptococcus parasanguinis*		
8	Abscess liver	*–*	*E. coli/Shigella *spp*.*	*Clostridium perfringens*
9	Abscess muscle	*P. micra*	*E. corrodens*	*F. nucleatum*
		*S. constellatus/intermedius*		*Porphyromonas circumdentaria*
10	Abscess ovarian	*Parvimonas *sp.	*–*	*F. nucleatum*
11	Abscess pancreas	*Enterococcus faecium *(*Bacteroides fragilis*)	*–*	*Bacteroides fragilis*
12	Abscess pancreas	*A. meyeri *(*P. melaninogenica*)	*Campylobacter concisus*	*P. melaninogenica/histicola*
				*Staphylococcus epidermidis*
13	Abscess para-aortic	*Corynebacterium tuberculostearicum*	*–*	*S. haemolyticus/epidermidis*
				*Corynebacterium appendicis*
14	Abscess psoas	*E. faecium*	*Enterobacter hormaechei*	*–*
15	Abscess spleen	*Enterococcus casseliflavus/gallinarum*	*E. coli/Shigella *spp*.*	*F. nucleatum*
		*Enterococcus faecalis*		*S. epidermidis*
		*Streptococcus anginosus*		
16	Abscess subcutaneous	*E. faecium*	*–*	(*E. faecium*)
		*Finegoldia magna*		(*F. magna*)
17	Abscess retroperitoneal	*E. faecium/durans*	*Klebsiella pneumoniae*	*–*
18	Bile	*–*	*E. coli/Shigella *spp*.*	*F. nucleatum*
19	Bile	*Enterococcus caccae*	*–*	*S. epidermidis*
		*E. casseliflavus/gallinarum*		
		*E. faecalis*		
20	Empyema	*P. micra*	*–*	*Prevotella nigrescens*
		*S. anginosus*		*Peptostreptococcus stomatis*
21	Empyema	*S. intermedius*	*C. gracilis*	*Fusobacterium *sp.
22	Empyema	*P. micra*	*C. gracilis*	*F. nucleatum*
		*S. intermedius*		
23	Empyema	*P. micra*	*C. gracilis*	*F. nucleatum*
		*S. constellatus*	*Haemophilus parainfluenzae*	*P. melaninogenica*
24	Empyema	*P. micra*	*C. gracilis*	*Prevotella pleuritidis*
		*S. intermedius*	*E. corrodens*	
25	Empyema	*Dialister invisus*	*–*	*P. nigrescens *†
		*Dialister micraerophilus*		*Prevotella veroralis*
		*P. micra*		

* Differentiation between *S. constellatus* and S. *intermedius* based on type strains only.

† Chromatogram too complex to allow for complete analysis. Only dominant peaks included.

Among the 25 clinical specimens assumed to be negative (Table 4) one sample (ID 35) was found to be positive by the universal PCR. It reached *C*_t_ at cycle 31.5, exactly three cycles before the negative control. Melt-point analysis showed no distinct peak and it was negative by the group-specific PCRs. Sequencing was unsuccessful and the PCR result was defined as a false positive. Another sample (ID 50) was positive by PCR ‘B’. This reached *C*_t_ at cycle 30.6, 3.1 cycles before the closest negative control. Melt-point analysis gave an irregular peak. Sequencing produced a complex chromatogram dominated by a *Pseudomonas* sp. and an *Acidovorax* sp. This corresponds to what we typically find in our negative controls (*Acidovorax* spp., *Acinetobacter* spp., *Comamonas* spp., *Pseudomonas* spp. and *Sphingomonas* spp.) and the PCR was judged to be a false positive. The remaining 98 PCRs were negative as expected.

**Table 4.** Results obtained for the presumably sterile samples by culture and by the two direct sequencing protocols

ID	Specimen	Culture	Universal PCR	Group-specific PCRs			Antibiotics*
				A	B	C	
26	Abscess liver (amoebic)	No growth	Neg	Neg	Neg	Neg	Unknown
27	Abscess lung (aspergilloma)	No growth	Neg	Neg	Neg	Neg	CT
28	Abscess neck (tumor colli)	No growth	Neg	Neg	Neg	Neg	–
29	Abscess pelvic (cancer)	No growth	Neg	Neg	Neg	Neg	Unknown
30	Abscess subcutaneous (cancer)	No growth	Neg	Neg	Neg	Neg	CF
31	Biopsy bone	No growth	Neg	Neg	Neg	Neg	–
32	Pericardial fluid	No growth	Neg	Neg	Neg	Neg	GE, PC
33	Pericardial fluid	No growth	Neg	Neg	Neg	Neg	AM, CI
34	Pericardial fluid	No growth	Neg	Neg	Neg	Neg	Unknown
35	Peritoneal fluid	No growth	***Pos***†	Neg	Neg	Neg	OX
36	Peritoneal fluid	No growth	Neg	Neg	Neg	Neg	–
37	Pleural fluid	No growth	Neg	Neg	Neg	Neg	–
38	Pleural fluid	No growth	Neg	Neg	Neg	Neg	–
39	Pleural fluid	No growth	Neg	Neg	Neg	Neg	–
40	Pleural fluid	No growth	Neg	Neg	Neg	Neg	–
41	Pleural fluid	No growth	Neg	Neg	Neg	Neg	–
42	Pleural fluid	No growth	Neg	Neg	Neg	Neg	Unknown
43	Subdural haematoma	No growth	Neg	Neg	Neg	Neg	–
44	Synovial fluid	No growth	Neg	Neg	Neg	Neg	Unknown
45	Synovial fluid	No growth	Neg	Neg	Neg	Neg	–
46	Synovial fluid	No growth	Neg	Neg	Neg	Neg	Unknown
47	Synovial fluid	No growth	Neg	Neg	Neg	Neg	–
48	Tissue prosthetic joint	No growth	Neg	Neg	Neg	Neg	Unknown
49	Tissue prosthetic joint	No growth	Neg	Neg	Neg	Neg	–
50	Tissue prosthetic joint	No growth	Neg	Neg	***Pos***‡	Neg	–

* Antibiotics: AM, amoxicillin; CI, ciprofloxacin; CF, cefalotin; CT, cefotaxime; GE, gentamicin; OX, oxacillin; PC, penicillin.

† Distance to negative control 3.0 cycles; *C*_t_ = 31.5.

‡ Distance to closest negative control 3.1 cycles; *C*_t_ = 30.6.

## Discussion

Detection and identification of bacteria directly from polymicrobial clinical samples by broad-range PCR followed by DNA sequencing can be accomplished using RipSeq mixed chromatogram analysis. In this study we replaced the standard universal 16S rRNA gene PCR with a set of three separate group-specific PCRs, reducing the risk of species present at lower concentrations becoming outcompeted in the amplification step. The importance of this factor is illustrated by the fact that only eight of the chromatograms obtained with the universal primers were judged to contain more than three bacterial sequences (Table 2).

The group-specific PCRs significantly increased the number of identified bacteria as compared to amplification with a universal PCR (95 versus 51, *P*<0.05 by Wilcoxon signed-rank test; Altman, 1991). All bacteria identified by standard universal direct sequencing and RipSeq analysis were also found with the group-specific primers and RipSeq analysis. Since the species combinations in the chromatograms obtained with the universal versus the group-specific primers for the most part were different, this is a confirmation of RipSeq’s ability to analyse mixed DNA chromatograms. In addition 18 of the bacteria identified as part of a mixed chromatogram with the universal primer pair were recovered as non-mixed chromatograms with one of the group-specific primer pairs.

Discrimination between the three groups of bacteria was based on the forward primers alone, and the difference between forward primers A and C was a single nucleotide at the 3′-terminal position only (Table 1). In order to obtain sufficient primer discrimination we used locked nucleic acids (LNAs). LNAs increase the impact of a mismatch in the actual and the subsequent base position. When placed in the penultimate 3′-end position an LNA protects the 3′-end terminal base from the proofreading 3′–5′-exonuclease activity of the TaKaRa polymerase. In combination with this primer design, polymerases without 3′–5′-exonuclease activity have been reported to provide less robust discrimination (Di Giusto & King, 2004; Rupp *et al.*, 2006). LNAs were also useful for harmonizing primer annealing temperatures.

In a set of group-specific primers the number of primer pairs will be a compromise between the possibilities given by the chosen gene target, sensitivity and practical considerations. For maximum effect, relevant bacterial species should distribute evenly between the primer pairs and cross-reactivity should be low. We found an almost perfect distribution for the species included in the pre-study alignments, and we also saw a good distribution in the study: 42 species were targeted by primer pair A, 20 by primer pair B and 33 by primer pair C. Given that two targets belong to different primer groups the primers described here will accommodate ratios of 1 : 1000 or higher. With two targets from the same group the situation will be equivalent to what we see with a single universal primer pair, where successful detection of both targets cannot be expected with ratios higher than 1 : 10.

A negative control containing a mixed DNA population in low concentration could give low reproducibility with the group-specific primers due to random distribution of the different DNA targets in the respective reaction tubes. We also considered that low-level DNA contamination in a specimen could have a different composition from low-level DNA contamination in the negative control (due to e.g. sample collection and transportation tubes). Consequently, in reactions where the negative control did not contain any target DNA a poor amplification would lead to a very high *C*_t_ value that subsequently could define a negative sample as positive. For these reasons, although the negative control was included in all group-specific PCRs, the PCR in which it became positive first defined the cut-off for a positive sample for all three reactions. With the definition of a positive PCR used in this study, we got two false-positive PCRs. Although none of the false positive PCRs resulted in findings that would have been reported, we suggest including a second criterion stating that in order to be defined as positive, a sample has to reach *C*_t_ before cycle 30 in addition to becoming positive three cycles before the negative control. In our experience reactions with higher *C*_t_ values are of doubtful significance and rarely result in relevant findings. This additional rule would not have affected the results in Tables 2 and 3.

By using group-specific primers the cost per analysis will see some increase. We kept the increase in both price and workload as low as possible by sequencing in one direction only, and the average number of sequencing reactions in our material was 2.55 per positive sample. There are several publications based on mono-directional sequencing, and it is also used in a commercially available 16S rDNA sequencing kit (Bosshard *et al.*, 2003, 2004; Wellinghausen *et al.*, 2009). With the high fidelity of today’s PCR and Sanger sequencing reactions the main reason for bidirectional sequencing is to obtain maximum read lengths and better discrimination between similar species. If we ignore the primer-binding areas which are of no value for identification, a reduction of 40–50 readable bases can be expected with monodirectional versus bidirectional sequencing. With our primers this led to a 40–50 % reduction in the number of readable bases in the variable area V3, whereas the variable areas V1 and V2 remained intact (Baker *et al.*, 2003). We find the potential reduction in resolution to the species level defendable when compared to the significant increase in identifiable bacteria. The only visible consequence of mono-directional sequencing in this study was found in sample 12, where the better discrimination between *Prevotella histicola* and *P. melaninogenica* in the forward direction allowed for an ‘and’ instead of an ‘and/or’ identification with the universal protocol. A rational approach will be to use a single universal primer pair for specimens expected to be monobacterial (e.g. cerebrospinal fluid and synovial fluid) and reserve the group-specific primers for specimens known to frequently contain multiple organisms (e.g. abscesses, pleural fluid and bile).

It is clear from this and other studies that the results achieved by routine culture-based diagnostics alone are not sufficient when it comes to patients who have received one or more doses of antibiotics prior to sample collection, or the investigation of anaerobe infections (Al Masalma *et al.*, 2009; Goldenberger *et al.*, 1997; Kommedal *et al.*, 2009; Petti *et al.*, 2008b; Schuurman *et al.*, 2004; Senn *et al.*, 2005). Infectious-disease physicians are well aware of these limitations. As a consequence microbiological results are currently not so often used to tailor individual antibiotic treatment, but more to look for the unexpected organisms that eventually should lead to a diversion from standard empirical therapy. Compared to culture, sequencing with group-specific primers detected on average 2.5 times as many species per sample including important pathogens such as *Actinomyces meyeri*, *Campylobacter gracilis* (de Vries *et al.*, 2008; Johnson *et al.*, 1985), *Escherichia coli*, *Fusobacterium nucleatum* and HACEK group bacteria (*Haemophilus parainfluenzae*, *Aggregatibacter aphrophilus* and *Eikenella corrodens*). Failure to detect these organisms can lead to inadequate antimicrobial coverage, in particular in situations where first-line empirical treatment cannot be used or when patients are to be transferred to oral treatment. For the brain abscesses our findings correlate well with what was described in a study by Al Masalma *et al.* (2009) using cloning and second-generation sequencing. Although these methods can provide even higher resolution in some situations, today they remain research tools due to long investigation time and high costs.

For the uncultured organisms there will be no antimicrobial susceptibility results to guide treatment. Since some of these species may be unfamiliar to the clinical doctors, adding comments on the general susceptibility patterns for these species to the lab reports may contribute to assuring adequate patient treatment and also to preventing unnecessary use of broad-spectrum antibiotics. Many of the species detected exclusively by sequencing in this study (e.g. the anaerobic bacteria) have relatively unproblematic susceptibility patterns.

The purposes of this study were to demonstrate the potential of the group-specific PCRs and to establish a robust protocol. The approach can contribute to better characterization of polybacterial clinical samples and has the advantage that it can be readily implemented in any diagnostic laboratory with experience in standard direct sequencing.

## Supplementary Material

Supplementary tables
