# Supplementary material for: Characterization of polybacterial clinical samples using a set of group-specific broad-range primers targeting the 16S rRNA gene followed by DNA sequencing and RipSeq analysis
Source: J Med Microbiol. 2011 Jul;60(Pt 7):927–36. doi: 10.1099/jmm.0.028373-0 (PMC3168215; doi:10.1099/jmm.0.028373-0)
Supplement: Supplementary tables [file supp_60_7_927__index.html]

Characterization of polybacterial clinical samples using a set of group-specific broad-range primers targeting the 16S rRNA gene followed by DNA sequencing and RipSeq analysis — Supplementary tables 

# Characterization of polybacterial clinical samples using a set of group-specific broad-range primers targeting the 16S rRNA gene followed by DNA sequencing and RipSeq analysis

### Characterization of polybacterial clinical samples using a set of group-specific broad-range primers targeting the 16S rRNA gene followed by DNA sequencing and RipSeq analysis, by Ø. Kommedal, K. Lekang, N. Langeland and H. G. Wiker

*Journal of Medical Microbiology* vol. **60**, part 7, pp. 927 - 936

**Table S1.** Distribution of species among primer groups [Excel file] (43 kb)   
  
**Table S2.** Primer cross-reactivity experiments with artificial bacterial DNA mixes [PDF] (48 kb)
