# Supplementary material for: Characterization of polybacterial clinical samples using a set of group-specific broad-range primers targeting the 16S rRNA gene followed by DNA sequencing and RipSeq analysis
Source: J Med Microbiol. 2011 Jul;60(Pt 7):927–36. doi: 10.1099/jmm.0.028373-0 (PMC3168215; doi:10.1099/jmm.0.028373-0)
Supplement: Supplementary tables [file supp_60.7.927_Table_S2.pdf]

Supplementary material for Kommendal *et al.* (2011), *J Med Microbiol* **60**, 927–936

Table S2. Primer cross-reactivity experiments with artificial bacterial DNA mixes

**Amplification with group A PCR**		
Relative DNA concentrations		Chromatogram analysis
Streptococcus pyogenes (plasmid group A)	*Staphylococcus aureus* (plasmid group C)	
1	1	*Streptococcus pyogenes*
1	10	*Streptococcus pyogenes*
1	100	*Streptococcus pyogenes*
1	1000	*Streptococcus pyogenes* + *Staphylococcus aureus* (equal peak heights)
Streptococcus pyogenes (plasmid group A)	*Escherichia coli* (plasmid group B)	
1	1	*Streptococcus pyogenes*
1	10	*Streptococcus pyogenes*
1	100	*Streptococcus pyogenes*
1	1000	*Streptococcus pyogenes*
**Amplification with group B PCR**		
Relative DNA concentrations		Chromatogram analysis
Klebsiella pneumoniae (genomic DNA group B)	*Staphylococcus aureus* (genomic DNA group C)	
1	1	*Klebsiella pneumoniae*
1	10	*Klebsiella pneumoniae*
1	100	*Klebsiella pneumoniae*
1	1000	*Klebsiella pneumoniae*
Klebsiella pneumoniae (genomic DNA group B)	*Enterococcus faecalis* (genomic DNA group A)	
1	1	*Klebsiella pneumoniae*
1	10	*Klebsiella pneumoniae*
1	100	*Klebsiella pneumoniae*
1	1000	*Klebsiella pneumoniae*
**Amplification with group C PCR**		
Relative DNA concentrations		Chromatogram analysis
Staphylococcus aureus (plasmid group C)	*Stenotrophomonas maltophilia* (plasmid group B)	
1	1	*Staphylococcus aureus*
1	10	*Staphylococcus aureus*
1	100	*Staphylococcus aureus*
1	1000	*Staphylococcus aureus*
Staphylococcus aureus (plasmid group C)	*Escherichia coli* (plasmid group B)	
1	1	*Staphylococcus aureus*
1	10	*Staphylococcus aureus*

1	100	*Staphylococcus aureus*<br>(dominant peaks)<br>+<br>*Enterococcus faecalis*<br>(low secondary peaks)
1	1000	*Staphylococcus aureus* +<br>*Enterococcus faecalis*<br>(equal peak heights)
